# Nogo-A-deficient Transgenic Rats Show Deficits in Higher Cognitive Functions, Decreased Anxiety, and Altered Circadian Activity Patterns

**DOI:** 10.3389/fnbeh.2014.00090

**Published:** 2014-03-18

**Authors:** Tomas Petrasek, Iva Prokopova, Martin Sladek, Kamila Weissova, Iveta Vojtechova, Stepan Bahnik, Anna Zemanova, Kai Schönig, Stefan Berger, Björn Tews, Dusan Bartsch, Martin E. Schwab, Alena Sumova, Ales Stuchlik

**Affiliations:** ^1^Department of Neurophysiology of Memory, Institute of Physiology, Academy of Sciences of the Czech Republic, Prague, Czech Republic; ^2^First Faculty of Medicine, Charles University in Prague, Prague, Czech Republic; ^3^Department of Neurohumoral Regulations, Institute of Physiology, Academy of Sciences of the Czech Republic, Prague, Czech Republic; ^4^Social Psychology, Department of Psychology II, University of Würzburg, Würzburg, Germany; ^5^Department of Molecular Biology, Central Institute of Mental Health, Mannheim, Germany; ^6^Brain Research Institute, University of Zurich, Zurich, Switzerland; ^7^Neurosciences, Department of Biology, Swiss Federal Institute of Technology Zurich, Zurich, Switzerland; ^8^Division of Molecular Mechanisms of Tumor Invasion, German Cancer Research Center, Heidelberg, Germany

**Keywords:** Nogo-A, AAPA, Carousel maze, passive avoidance, neophobia, anhedonia, circadian rhythmicity

## Abstract

Decreased levels of Nogo-A-dependent signaling have been shown to affect behavior and cognitive functions. In Nogo-A knockout and knockdown laboratory rodents, behavioral alterations were observed, possibly corresponding with human neuropsychiatric diseases of neurodevelopmental origin, particularly schizophrenia. This study offers further insight into behavioral manifestations of Nogo-A knockdown in laboratory rats, focusing on spatial and non-spatial cognition, anxiety levels, circadian rhythmicity, and activity patterns. Demonstrated is an impairment of cognitive functions and behavioral flexibility in a spatial active avoidance task, while non-spatial memory in a step-through avoidance task was spared. No signs of anhedonia, typical for schizophrenic patients, were observed in the animals. Some measures indicated lower anxiety levels in the Nogo-A-deficient group. Circadian rhythmicity in locomotor activity was preserved in the Nogo-A knockout rats and their circadian period (tau) did not differ from controls. However, daily activity patterns were slightly altered in the knockdown animals. We conclude that a reduction of Nogo-A levels induces changes in CNS development, manifested as subtle alterations in cognitive functions, emotionality, and activity patterns.

## Introduction

The protein Nogo-A, belonging to the Reticulon family of proteins, is an important member of the class of myelin-associated inhibitors of axonal growth. It is present principally in the oligodendrocytes, but is expressed by some neuron subpopulations as well. When exposed in the cellular membrane, the Nogo-A molecule acts via two principal receptors, Nogo-66 receptor (NgR) and paired immunoglobulin-like receptor B (PirB) (see Schwab, 2010 for review).

The Nogo-A is widely recognized for its relevance in various physiological and pathological processes. The protein was originally noted as an inhibitor blocking axonal regrowth and plasticity after CNS injuries (Chen et al., 2000; GrandPré et al., 2000; Schwab, 2004). Subsequently, the Nogo-A-dependent signaling has shown to be crucial in the development and migration of neurons (Mingorance et al., 2004; Mingorance-Le Meur et al., 2007) and glial cells (Pernet et al., 2008; Chong et al., 2012). In the adult brain, Nogo-A (especially the neuronal Nogo-A) contributes to the modulation of neuronal and synaptic plasticity (Akbik et al., 2012; Pernet and Schwab, 2012) and adult neurogenesis (Rolando et al., 2012).

It is therefore not surprising that the disruption of a Nogo signaling pathway in the developing brain has been suggested to play a role in neuropsychiatric diseases of neurodevelopmental origin, most notably schizophrenia and bipolar disorder (Willi and Schwab, 2013). This view is corroborated by a reported genetic linkage between chromosomal loci for the Nogo-A or its receptor and susceptibility to schizophrenia (Novak et al., 2002; Sinibaldi et al., 2004; Tan et al., 2005; Hsu et al., 2007; Budel et al., 2008; Voineskos, 2009; Jitoku et al., 2011). Schizophrenia is characterized by abnormal development and function of the hippocampus (Harrison, 2004). The hippocampus is a prime example of a structure exhibiting a high degree of neuronal and synaptic plasticity, as well as the neuronal expression of Nogo-A persisting well into adulthood. Therefore, it should be particularly liable to pathophysiological processes disrupting Nogo-A-dependent signaling, or corresponding experimental manipulations. The study was aimed to elucidate the behavioral effects of decreased Nogo-A expression on behavior, with a focus on hippocampal function and schizophrenia-like endophenotypes. We took advantage of a novel animal model, a Nogo-A knockdown rat exhibiting decreased expression of the Nogo-A protein in the brain tissue, most notably in neurons (Tews et al., 2013).

Apart from other places in the brain, Nogo-A has been found to be expressed in subsets of neurons of the retina (Huber et al., 2002; Wang et al., 2002; Hunt et al., 2003), which is a part of the internal time keeping circadian system (for review, see Meijer and Schwartz, 2003). Moreover, the function of the central circadian clock, located in the suprachiasmatic nuclei (SCN) of the hypothalamus, is modulated by neuronal connections with other brain areas including the hippocampus (Canteras and Swanson, 1992), and, vice versa, the circadian clock can affect hippocampal functions such as long-term memory formation (Stephan and Kovacevic, 1978; Tapp and Holloway, 1981). Therefore, we also focused on an analysis of basic circadian properties of the central clock in the SCN of Nogo-A knockdown rats, using daily rhythm in locomotor activity as a direct output of the clock.

We hypothesized that the animals should exhibit impairment in the active place avoidance task on the Carousel maze, which is highly sensitive in impairments of hippocampal function (Cimadevilla et al., 2001), and has been successfully employed in evaluation of animal models of schizophrenia (Stuchlik et al., 2004; Vales et al., 2006; Bubenikova-Valesova et al., 2008a). We also expect abnormal rhythmicity patterns resulting from changes in the circadian systems.

## Materials and Methods

### Transgenic model

The transgenic rat model was prepared in the Central Institute of Mental Health (CIMH, Mannheim, Germany), in cooperation with Martin Schwab from the Brain Research Institute, University of Zurich and Department of Health Science and Technology, Swiss Federal Institute of Technology (ETH) Zurich, on the genetic background of the Sprague-Dawley rat. The particular transgenic line used in this study is designated as SD-Tg(CAG-RNAi: Nogo-A, EGFP)L2ZI, short L2; standing for line 2, and is of outbred genetic background. The parental subjects were obtained from Charles River, Germany.

The expression of Nogo-A was suppressed by means of the insertion of a genetic construct expressing a small interfering RNA, complementary to Nogo-A mRNA (targeting Nogo-A-specific exon 3 of *Rtn4*), binding to it with a high affinity and therefore preventing translation. Because the blockade is not total, the levels of neuronal Nogo-A were reduced to about 50% in the CNS as a whole, 30% in the cerebral cortex, and 60% in the hippocampus. Nogo-A levels in the oligodendrocytes were affected to a lesser extent relative to neurons (Tews et al., 2013).

The knockdown manifests itself on the cellular level by increased long-term potentiation, and leads to behavioral abnormalities as well (Tews et al., 2013). This resembles the schizophrenia-like behavior noted previously in knockout mice (Willi et al., 2010). Subtle cognitive deficit has been described in Petrasek et al. (2014). The distribution pattern of biochemical markers in the brains of the transgenic rats also paralleled some changes observed in human schizophrenic patients (Krištofiková et al., 2013).

### Animals

Male Nogo-A knockdown rats from two different litters and non-littermate, age-matched, and wild-type (WT) Sprague-Dawley controls obtained from the breeding colony of the CIMH, Mannheim, Germany, were used. After arrival at the Institute of Physiology, an appropriate acclimatization period (1 month) followed before the start of experimental procedures, and all the animals were accustomed to the experimenters during 1 week of daily handling. The rats were housed in groups of two or three in an air-conditioned animal room, with free access to food and water. The animals were kept on 12/12 light–dark cycle, and the experiments were performed during the light phase. The rats were 5 months old when the testing started (Carousel maze) and about 8 months old when sacrificed, their weights were between 540 and 750 g. For time schedule of the behavioral experiments, see Table 1.

**Table 1:** **Time schedule of behavioral experiments**.

Age (months)	5.3	5.5		5.7	5.9			6.1	6.3	6.5	6.7	6.9	7.1	7.3	7.5	7.7	7.9	7.9	8.0
Weeks	1	2		3	4			5	6	7	8	9	10	11	12	13	14	15	16
Task	Carousel maze				Step-through avoidance			Beam walking	Neophobia/anhedonia			Circadian rhythmicity							Sacrifice
	
	
	Habituation	Acquisition	Retrieval	Reversal	Habituation	Acquisition	Test					12/12 LD cycle			Constant darkness				

	
Number of daily sessions	5	5	1	5	2	1	1	1	2			29			16				

The original group included nine Nogo-A knockdown and nine control animals, however, some individuals died before the completion of the experiments, therefore the group size was diminished in neophobia/anhedonia (eight Nogo-A knockdown rats and nine controls) and circadian rhythmicity tests (five Nogo-A knockdown rats and eight controls).

All animal experimentation complied with the Animal Protection Code of the Czech Republic and international guidelines including EU directives (2010/63/EC).

### RNA isolation and real-time qRT-PCR

After completion of behavioral experiments, the rats were sacrificed by cervical dislocation, and hippocampal and cerebellar samples were removed and stored in RNA Later (Sigma, USA) at −80°C until processed. Total RNA was extracted by homogenization from the cerebellum, left and right hippocampus of five Nogo-A knockdown and eight control rats and subsequently purified using the RNeasy Mini kit (Qiagen, USA) according to the manufacturer’s instructions. RNA concentrations were determined by spectrophotometry at 260 nm, and the RNA quality was assessed by electrophoresis on a 1.5% agarose gel. Moreover, the integrity of randomly selected samples of total RNA was tested using an Agilent 2100 Bioanalyzer (Agilent Technologies, USA).

The qRT-PCR method used to detect Nogo-A mRNA has been described previously (Sládek et al., 2007). Briefly, 1 μg of total RNA was reverse transcribed using the SuperScript VILO cDNA synthesis kit (Life Technologies, USA) with random primers. The resulting cDNAs were used as templates for qRT-PCR. Diluted cDNA was amplified on LightCycler 480 (Roche, Switzerland) using the Express SYBR GreenER qPCR SuperMix (Life Technologies, USA) and the corresponding primers for Nogo-A (forward 5′-CAG TGG ATG AGA CCC TTT TTG-3′, reverse 5′-GCT GCT CCA TCA AAT CCA TAA-3′) or GFP (forward 5′-CAA CAG CCA CAA CGT CTA TAT CAT-3′, reverse 5′-ATG TTG TGG CGG ATC TTG AAG-3′). Relative quantification was achieved using a standard curve and subsequently normalizing the gene expression to β2-microglobulin (B2M, forward 5′-TCT CAC TGA CCG GCC TGT ATG CTA TC-3′, reverse 5′-AAT GTG AGG CGG GTG GAA CTG TG-3′), which has been used as a housekeeping gene previously (Sládek et al., 2007). Its expression was stable throughout the day and did not vary among the analyzed tissues.

### Protein isolation and western blot

Samples of the left hippocampus (50–100 mg) from five Nogo-A knockdown and three control animals were homogenized in 1 ml of CelLytic MT extraction reagent (Sigma, USA) according to the manufacturer’s protocol. Protein concentration was determined by Bradford assay (Thermo Scientific, USA). All reagents for Western blot were purchased from Life Technologies, USA, unless stated otherwise. The hippocampal homogenate (21 μg of total protein) was mixed with a NuPAGE LDS Sample buffer and Sample reducing reagent, denatured at 70°C for 10 min, and separated with a protein ladder on a NuPAGE Tris–Acetate premade gel according to the manufacturer’s instructions using a NuPAGE running buffer with an antioxidant. The protein was transferred by electro-blotting in a NuPAGE transfer buffer with 10% methanol onto a nitrocellulose membrane according to the manufacturer’s instructions. The membrane was blocked in a Western Blocker solution (Sigma, USA) for 1 h and then incubated with a primary antibody against Nogo-A H300 (Santa Cruz, USA) diluted 1:2000 in a Western Blocker overnight at 4°C on a rocker. The membrane was then washed five times for 5 min in TBST (2.42 g Tris–HCl, 8 g NaCl, 1 ml Tween 20 in 1000 ml of redistilled water, pH 7.6) and incubated with a secondary anti-rabbit HRP-conjugated antibody (Promega, USA) diluted 1:40,000 in a Western Blocker at room temperature (RT) for 1 h on a rocker. The membrane was then washed 5× in TBST, incubated with a SuperSignal West Pico Chemiluminescent substrate (Pierce, USA), and immunoreactive bands were detected after 45-s exposure using a cooled camera system. The membrane was subsequently incubated for 30 min at 50°C in a stripping buffer (31.25 ml of 1 M Tris–HCl, 10 g SDS, 3.5 ml of 100 mM 2-Mercaptoethanol in 500 ml of redistilled water), washed 5× in TBST, blocked in a Western Blocker for 1 h, incubated with an anti-actin 20–33 antibody (Sigma, USA) 1:350 in a Western Blocker for 1 h at RT, washed 5× in TBST, incubated with a secondary anti-rabbit HRP-conjugated antibody (Promega, USA) diluted 1:20,000 in a Western Blocker at RT for 1 h, washed 5× in TBST, incubated with a West Pico substrate, and exposed for 3 s. Photographs of the blots were imported into ImageJ (NIH, USA) software, where the optical density of individual lanes of detected Nogo-A protein was quantified relative to the actin internal standard.

### Behavioral tests

#### Carousel maze

The Carousel maze (for detailed description, see Stuchlik, 2007) consisted of a smooth featureless metallic circular arena (82 cm in diameter), enclosed by a 30-cm high transparent Plexiglas wall, and elevated 1 m above the floor of a 4 m × 5 m room containing an abundance of extra-maze cues. The behavior of the animals was recorded by a computer-based tracking system (Tracker, Biosignal Group, USA).

The active allothetic place avoidance (AAPA) task in the Carousel maze was employed, where the animals learned to avoid an unmarked sector, entrances into which were punished by mild electric shocks. The shock lasted 0.5 s, and was repeated after 1.5 s if the animal did not leave the sector. The intensity of current was individually adjusted for each rat to elicit escape reaction, ranging between 0.4 and 0.7 mA (50 Hz). There were no systematic differences in the shock levels between groups. The sector position was fixed in the reference frame of the room, so that the animals had to rely on extra-maze cues. Intra-maze cues (e.g., scent marks) were made unreliable by rotation of the arena, and the animals had to ignore these to solve the task. They also had to actively avoid the sector position, not to be transported there by movement of the arena.

The rats (tested at the age of 5–6 months) were trained during the light phase of the day, between 9:00 and 16:00 hours. Each daily session lasted for 20 min. The training consisted of three phases, each lasting 5 days: habituation (exploration of the whole apparatus without punishment), avoidance learning (acquisition), and training with a changed sector position (reversal). A single 5-min retrieval session (without shocks) was scheduled 24 h after the end of the acquisition phase, before onset of reversal training. No food deprivation or pellet chasing was involved.

To master this task, the animals need navigation skills and spatial memory to locate the to-be-avoided sector (which is directly imperceptible), as well as the ability to separate the landmarks into coherent representations and choose the relevant one. Separation of spatial frames has been suggested as an animal model of cognitive coordination (Wesierska et al., 2005), which is impaired in schizophrenic patients (Phillips and Silverstein, 2003), making the AAPA task very important in the study of animal models of schizophrenia.

This paradigm was similar, but simpler than the testing battery described in Petrasek et al. (2014), and was chosen for easier comparison with a large body of experimental results gained with the AAPA task.

#### Step-through avoidance

In this experiment, the same group of rats was used as in the Carousel maze experiment (7–8 months old during testing), with *n* = 9 for Nogo-A knockdown rats and *n* = 9 for controls. The step-through avoidance took place in an apparatus consisting of two compartments, one of which was open and brightly lit (1500 lx), while the second remained dark (<10 lx). Rats have a natural tendency to prefer the dark environment to the light, so they usually left the light compartment, where they were initially placed, and entered the dark half of the apparatus. After the entrance, the door between the compartments was always shut. The latency to step-through the door between compartments was recorded. During the two habituation trials, the entrance was neither rewarded nor punished. In the third, acquisition trial, however, a foot-shock (1.5 mA) was applied after entrance into the dark compartment. Individuals failing to enter the dark compartment altogether on this trial (lasting 5 min) were excluded from the experiment. After a 24-h delay, a testing session followed, during which the rats were exposed to the environment again. Increased latency to enter the dark compartment reflected memory from the previous experience.

#### Neophobia/anhedonia

The same group of rats was used in this experiment as in the previous ones, with *n* = 8 for Nogo-A knockdown rats and *n* = 9 for controls. Each rat, deprived of drinking water and food for 22 h, was put into a box containing two drinking bottles. One of them contained drinking water and the other was filled with saccharin solution (concentration 0.2%). After 60 min, the session was terminated and both bottles weighed to measure the amount of pure and sweetened water consumed. After this initial experience, the session was repeated after 24 h. The first session should elicit a conflict between preference for the sweet taste and the avoidance of the unfamiliar (neophobia), as the rats had never tasted saccharin before. In the second session, the rats were already familiar with the saccharin solution, and the amount consumed should reflect their taste preference. Failure to prefer the sweet taste could be taken as a sign of anhedonia, inability to enjoy a pleasant experience, observed in schizophrenia and depression (Pelizza and Ferrari, 2009) or in their animal models (Le Pen et al., 2002).

#### Beam walking test

The beam walking test was used to assess motor coordination of the same group of rats. The test requires a subject to cross a 2-m-long wooden beam that leads to a home-cage. Latency to reach the home-cage and number of slips and falls are measured to assess motor coordination. The results were already described in a different publication (Petrasek et al., 2014); therefore, we will not report them in detail in the present paper. Briefly, we did not find any difference between groups in any of the measured parameters.

### Monitoring of circadian locomotor activity

Nogo-A knockdown and WT Sprague-Dawley rats were kept in a 12-h of light and 12 h of darkness cycle (LD12:12) with free access to food and water for 29 days and then in constant darkness (DD) for 16 days. The rats were monitored for spontaneous activity during the entire protocol. To monitor locomotor activity, rats of both genotypes were kept individually in cages equipped with infrared movement detectors that were attached above the center of the cage top. A circadian activity monitoring system (Dr. H. M. Cooper, INSERM, France) was used to measure activity every minute, and double-plotted actograms were generated to evaluate the activity. The resulting data were analyzed using the ClockLab toolbox (Actimetrics, USA).

### Measured parameters and statistical design

#### mRNA and protein levels

The differences in mRNA and protein levels between both rat genotypes were evaluated by a Student’s *t*-test.

#### Carousel maze

The Carousel maze performance was evaluated using the track analysis program CM Manager 0.3.5 (Bahnik, 2013). Several behavioral measures were assessed. *Total distance* measured overall path traveled by a rat during a session, and was computed as a sum of linear distances between points selected every 1 s. Total distance can suggest deficits in locomotion or reveal rats that did not actively leave the to-be-avoided sector. *Maximum time avoided* was defined as the longest continuous time interval spent without an entrance into the to-be-avoided sector and was used as a measure of avoidance ability. *Mean distance from the center* of the arena was used as a measure of thigmotaxis. *Defecation* was assessed by counting the number of feces left on the arena floor after each session, serving as an additional measure of anxiety level. All these measures were used for analysis of performance during the acquisition and reversal phases. Furthermore, total distance, mean distance from the center, and defecation were used to assess locomotion, thigmotaxis, and anxiety during the habituation phase. Additionally, the *proportion of time spent in the opposite sector* was used to distinguish different behavioral strategies by assessing the time spent in the sector located 180° from the to-be-avoided sector. This measure was computed as a proportion of time spent in the opposite sector to the total time not spent in the to-be-avoided sector. We did not include time spent in the to-be-avoided sector in the denominator, otherwise the measure would be dependent on the avoidance ability. This measure was introduced for evaluating the reversal phase of the experiment, because the opposite sector during the reversal phase was in the same place as the to-be-avoided sector during the acquisition phase (the width of both sectors was the same, i.e., 60°). This measure was used to evaluate perseverance, which was observed in Nogo-A knockdown rats (and knockout mice) in some previous studies.

The parameters for acquisition and reversal phases were averaged across sessions before analysis. This was done because some values (approximately 0.6%) were missing for various reasons (e.g., tracking problems). Computing the averages enabled us to include all rats in the analysis. Before the averaging, we standardized values for each session. This was done because performance changed during subsequent days, and therefore the averages would be otherwise dependent on days when rats had missing values. To simplify comparison between phases, the averages were standardized again. Thus, we obtained one value for each rat for each of the two phases and every parameter. All parameters were then analyzed with mixed analysis of variance (ANOVA) with group (Nogo-A knockdown or control) as a between-subject factor and phase (acquisition and reversal) and as a within-subject factor. Since the mean value of all subjects for both phases was always zero due to the final standardization, the phase factor was included only to test for the interaction of group and phase factors (we used a similar analysis previously, Prokopova et al., 2012; Petrasek et al., 2014). Analyses for the habituation phase and for proportion of time spent in the opposite sector in the reversal phase were done in a similar manner, but the group comparisons were done with a Welch’s *t*-test. Correlations between used parameters are displayed in Table 2.

**Table 2:** **Correlations between measured parameters in the AAPA task**.

	Maximum time avoided – acquisition	Maximum time avoided – reversal	Distance from center – habituation	Distance from center – acquisition	Distance from center – reversal	Total distance – habituation	Total distance – acquisition	Total distance – reversal	Defecation – habituation	Defecation – acquisition	Defecation – reversal	Time in opposite – reversal
Maximum time avoided – acquisition												
Maximum time avoided – reversal	0.32											
Distance from center – habituation	− 0.50	− 0.01	
Distance from center – acquisition	−0.62*	−0.11	0.54	
Distance from center – reversal	−0.68*	−0.31	0.57*	0.91**	
Total distance – habituation	0.36	0.38	−0.72*	−0.35	−0.52	
Total distance – acquisition	0.90*	0.51	−0.56	−0.66*	−0.67*	0.66*						
Total distance – reversal	0.79	0.80	−0.31	−0.17	−0.29	0.27	0.55					
Defecation – habituation	−0.03	−0.39	−0.17	0.03	−0.02	−0.18	−0.07	−0.60				
Defecation – acquisition	0.19	0.07	0.02	−0.26	−0.14	−0.40	0.07	0.09	0.16			
Defecation – reversal	0.12	−0.20	−0.20	−0.36	−0.12	0.06	0.41	−0.16	0.46	0.59*		
Time in opposite – reversal	0.67	0.79*	−0.35	−0.24	−0.37	0.58	0.75**	0.61*	−0.18	−0.11	0.00	

The table shows correlations between all parameters that were used for analysis of the AAPA task. Generally, there are large correlations between the same parameters during different phases. Additionally, total distance, maximum time avoided, and proportion of time spent in the opposite sector are positively correlated with each other and negatively with mean distance from the center. Defecation does not appear to be reliably correlated with any other measure. The correlations were computed for both groups separately and pooled thereafter. *p < 0.05; **p < 0.01.

As it is shown in the results, three rats from the control group exhibited prolonged periods of immobility during the testing sessions and were not able to learn avoidance of the to-be-avoided sector. Therefore, we describe two analyses – one included all subjects from both groups, and the other excluded the three rats that were not able to learn the avoidance along with three rats from Nogo-A knockdown group that had the worst performance as determined by mean maximum time avoided from both phases (exclusion of these subjects had to be done, otherwise a possible difference between groups could have been explained by a selective exclusion of subjects from control group). Because the performance of immobile rats is not related to their cognitive abilities in any way, we believe that the analysis with exclusion of those subjects may better reveal differences between the two groups. The exclusion was not necessary for the habituation phase because no avoidance was needed during this phase. Note that after the exclusion, it was no longer true that the means for both phases had to be equal to zero, however, the phase factor would assess only in which phase were the excluded subjects relatively better, and therefore it was of no interest and its analysis is not reported in the Section “Results.”

Since values for three subjects were missing for retrieval phase and two other rats did not learn to avoid the to-be-avoided sector (as described above and in the Results), we did not perform any analysis for the retrieval phase. The number of subjects with valid values was low, and hence the low resulting statistical power would not enable us to find any effect other than extreme, which we did not expect.

All analyses were done with an R version 3.0.1 (R Core Team, 2013), which is also true for the step-through avoidance and neophobia/anhedonia tasks. Effect sizes are reported with generalized eta squared (Bakeman, 2005) or with a correlation coefficient.

#### Step-through avoidance

For the comparison of time it took subjects to move into the dark compartment during habituation and acquisition trials, we used a mixed ANOVA where group (Nogo-A knockdown or control) served as a between-subject factor and trial (two habituation trials and an acquisition trial were ordered and analyzed together) served as a within-subject factor. Polynomial contrasts were used for the trial factor. Because all but one subject remained in the light compartment during the entire testing session, the analysis was not deemed necessary for the testing session.

#### Neophobia/anhedonia

To compare the amount of saccharin consumed by both groups, we used a mixed ANOVA with group (Nogo-A knockdown or control) as a between-subject factor and session as a within-subject factor. Since the total amount of liquid consumed by both groups somewhat differed, we used a ratio of consumed saccharin solution to total amount of liquid consumed as a dependent variable.

#### Circadian rhythmicity

The differences in locomotor activity (i.e., the values of the total 24 h-activity and activity/rest ratio) between both rat genotypes were evaluated by a Student’s *t*-test, while difference in period was analyzed by the Mann–Whitney test. The analysis was done in Prism 6 software (Graphpad, USA).

## Results

### Confirmation of Nogo-A knockdown genotype

**Figure 1:**
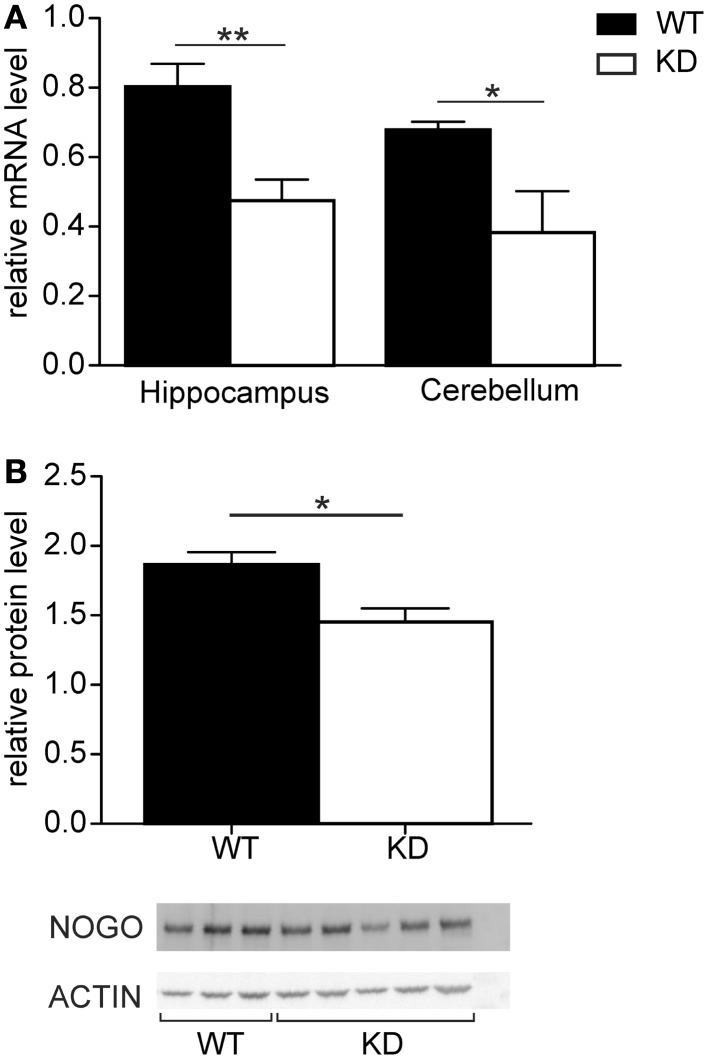
**Nogo-A mRNA and protein expression in Nogo-A knockdown rats (designated KD) and wild-type (WT) controls**. **(A)** Relative levels of Nogo-A mRNA in the hippocampus (*n* = 14 for WT, *n* = 10 for KD) and cerebellum (*n* = 7 for WT, *n* = 5 for KD) of wild-type (black) and knockdown (white) animals. **(B)** Relative levels of Nogo-A protein in the hippocampus of wild-type (black) and knockdown (white) animals. The graph shows mean optical density of Nogo-A normalized to actin of three WT and five KD animals.

The relative expression levels of Nogo-A were compared in the hippocampus and cerebellum of Nogo-A knockdown rats and Sprague-Dawley controls (Figure 1). In both tissues, the levels of Nogo-A mRNA were significantly reduced in the Nogo-A knockdown rats compared with controls [hippocampus *t*(22) = 3.52, *p* = 0.002, *r* = 0.60; cerebellum *t*(10) = 2.90, *p* = 0.02, *r* = 0.68; Student’s *t*-test]. The reduction was by about 40.9 ± 7.5% (mean ± SEM) in the hippocampus and 43.6 ± 17.5% in the cerebellum. Apart from mRNA, protein levels were also compared in the hippocampus of the Nogo-A knockdown and WT rats (Figure 1). The Nogo-A protein levels were significantly reduced in Nogo-A knockdown compared with WT rats by about 22.1 ± 5.5% [*t*(6) = 2.83, *p* = 0.03, *r* = 0.76; Student’s *t*-test].

### Carousel maze

**Figure 2:**
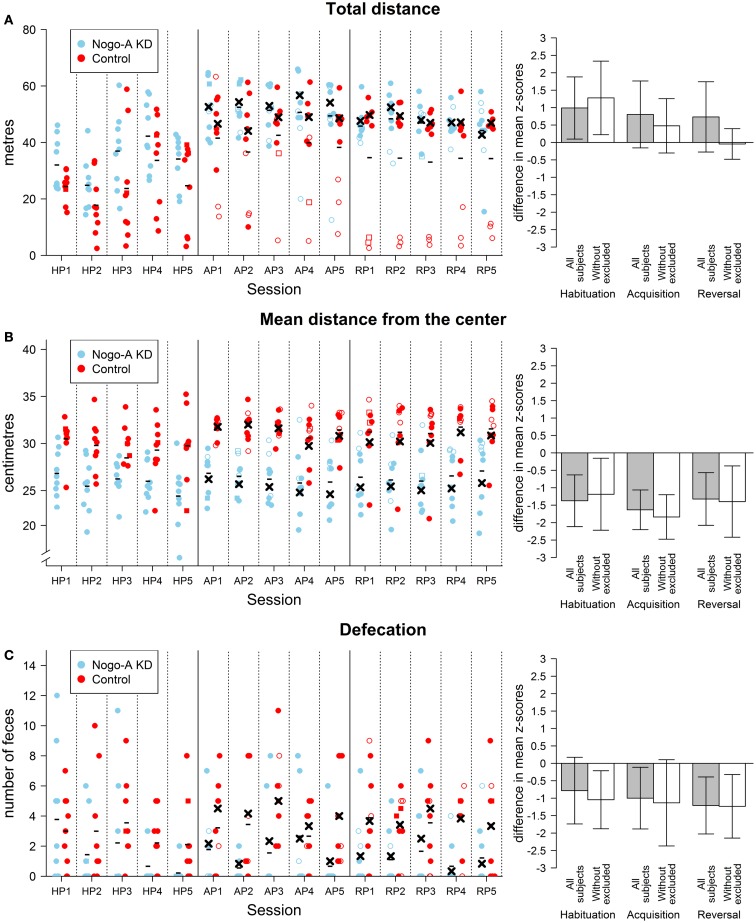
**Results of non-cognitive parameters in the AAPA task**. The plots on the left show individual values for each rat during every session. The sessions are labeled by HP, habituation phase; AP, acquisition phase; RP, reversal phase. Missing values are indicated by square points. These values were imputed by the closest value from the same phase for a given subject. When the missing value was in the middle of the phase and values for both adjacent sessions were valid, the missing value was imputed by their average. Missing values were not imputed and used in the analysis described in the text. For the acquisition and reversal phases, empty points indicate values for subjects that were excluded from analysis (see text for details). Minus signs indicate mean group values for a given session computed from all subjects and crosses indicate mean values computed from values without the excluded subjects. The means are computed from values including the imputed. For the habituation phase, no subjects were excluded from the analyses; therefore, only mean values computed for all subjects in a group are depicted. The bar plots on the right show differences between groups in the AAPA task for means of parameters averaged across sessions within a single phase. The differences are positive if Nogo-A knockdown group had higher values than control group. For every phase, both differences with and without excluded subjects are shown. Error bars show 95% confidence intervals of differences of means. Therefore, the error bars indicate whether there was a significant difference between groups for a given phase (which happens when the range within error bars does not include zero). However, they cannot be used for comparison between phases because they are not adjusted for dependency on the data. Furthermore, their use for comparison of the analyses with and without excluded subject is not meaningful as well. Even though the analysis of the habituation phase with the exclusion of subjects is not reported in text because the subjects could not show shock-induced freezing behavior during this phase, the differences with exclusion are depicted for this phase. **(A)** Results for total distance (left plot in meters). A difference between groups can be seen in all sessions in the habituation phase with Nogo-A knockdown rats displaying higher locomotion. The difference is still present during the subsequent phases, but only when values for all rats are compared. The difference in these phases is therefore mainly due to the rats in control group that did not actively avoid the to-be-avoided sector (i.e., rats depicted by empty points in the left plot). It can be seen that these rats generally moved <20 m during a session and they were even less active during the reversal phase. It can be observed that one of these rats moved in a degree comparable to other rats at the beginning of the acquisition phase but its locomotion hugely decreased in subsequent sessions. **(B)** Results for mean distance from the center (left plot in centimeters). A large difference between groups can be seen for mean distance from the center in all phases and sessions. Furthermore, the difference is not dependent on the exclusion of the subjects. Nogo-A knockdown rats tended to be present closer to the center of the arena than rats from control group. Note that the *y*-axis in the left plot does not start at zero. Arena diameter is 82 cm, therefore the maximum theoretically possible value of mean distance from the center is somewhat lower than 41 cm, which would correspond to being next to the margin of the arena during the entire session. **(C)** Results for defecation. Defecation was lower in Nogo-A knockdown rats in all three phases. However, the difference was not significant in the habituation phase. There appears to be little difference between the results of the analyses done with and without the excluded rats.

Visual observation showed normal behavior during the habituation phase and rapid acquisition of the task in the majority of the rats. Three individuals from the control group, however, exhibited marked immobility during avoidance sessions. We assume that these animals adopted passive behavior (freezing) instead of an active approach (escape) as a reaction to the aversive nature of the task. Active locomotion is a basic prerequisite for the successful mastering of this task, therefore, the animals exhibiting freezing as the dominant strategy were excluded as “non-solvers” along with three other rats from the Nogo-A knockdown group for the reasons explained in the Section “Measured Parameters and Statistical Design.” The visual observation was supported by values of the non-solvers for total distance parameter. We computed standardized averages of total distance for each rat for acquisition and reversal sessions as described in the Section “Measured Parameters and Statistical Design.” We then averaged these values and standardized them again. The three rats displaying freezing behavior had *z*-scores for the resultant total distance parameter −2.7, −1.9, and −1.2 while all other rats had values in a range from −0.4 to 1.0 (values for total distance are shown in Figure 2A). Additionally, the freezing behavior can be seen in the subject’s response to obtaining a shock. We used the median absolute speed after shock to assess this response. This parameter is computed as a median of angular speed during 1 s following a shock. Since subjects can move outside of the sector both by moving with or against the direction of rotation of the arena, we used absolute speeds to assess whether administration of a shock elicited a response. While all other rats showed some active response (mean median absolute speed after shock higher than 8°/s), the three non-solvers displayed no such behavior (mean median absolute speed after shock lower than 2°/s).

Results for total distance from the habituation phase showed a significant difference between the two groups, *t*(14.93) = 2.37, *p* = 0.03, *r* = 0.51, with the control group having a lower total distance than the Nogo-A knockdown group. Additionally, there was a significant difference in the mean distance from the center, *t*(13.15) = −3.99, *p* = 0.002, *r* = −0.71. Nogo-A knockdown rats preferred positions closer to the center of arena than rats from the control group. Defecation was higher in the control group, but not significant, *t*(13.51) = −1.76, *p* = 0.10, *r* = −0.40.

**Figure 3:**
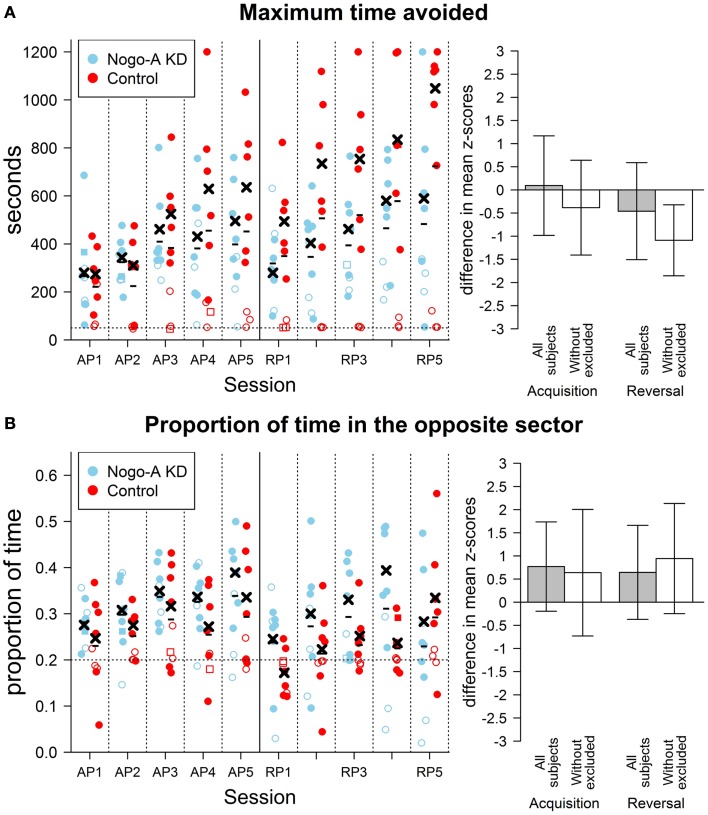
**Results of cognitive parameters in the AAPA task**. The plots on the left show individual values for each rat during both sessions. The sessions are labeled by abbreviations: AP, acquisition phase; RP, reversal phase. Missing values are indicated by square points. Empty points indicate values for subjects that were excluded from analysis (see text for details). Minus signs indicate mean group values for a given session computed from all subjects and crosses indicate mean values computed from values without the excluded subjects. The bar plots on the right show differences between groups in the AAPA task for means of parameters averaged across sessions within a single phase. The differences are positive if Nogo-A knockdown group had higher values than control group. For both phases, both difference with and without excluded subjects is shown. Error bars show 95% confidence intervals of differences of means. See description of the Figure 2 for further details. **(A)** Results for maximum time avoided (left plot in seconds). No difference between groups is visible when all subjects are compared. However, a significant difference can be seen when the comparison is done without the excluded rats. Note, especially, the large difference in means for the last few session of the reversal phase. The horizontal dotted line in the left plot represents 50 s, which corresponds to a time of maximum time avoided for a subject that is fully immobile. The excluded rats from control group do not usually show better avoidance ability than they would have if they did not move at all. Maximum possible value for maximum time avoided is 1200 s, which corresponds to no entrance to the to-be-avoided sector. **(B)** Results for proportion of time in the opposite sector. A slight, but not significant, increase in the proportion of time in the opposite sector can be seen in Nogo-A knockdown rats in all sessions with exception of the last one. The result is contrary to that expected based on previous research suggesting perseverance in Nogo-A knockdown animals. The horizontal dotted line in the left plot depicts a proportion that would be measured for totally immobile subjects. It can be seen that the three excluded rats from Nogo-A knockdown group have values close to this line. The acquisition phase was not included in analysis and is shown only for comparison.

The analysis of maximum time avoided (Figure 3A) for acquisition and reversal phases with all subjects included revealed no effect of group, *F*(1, 16) < 1, *p* = 0.67, $\eta_{G}^{2}=0.01,$ and no interaction between group and phase factors, *F*(1, 16) = 1.41, *p* = 0.25, $\eta_{G}^{2}=0.02.$ For total distance, we found a marginally significant effect of group, with the control group having lower total distance than the Nogo-A knockdown group, *F*(1, 16) = 3.55, *p* = 0.08, $\eta_{G}^{2}=0.16,$ and no interaction of group and phase factors, *F*(1, 16) < 1, *p* = 0.85, $\eta_{G}^{2}=0.00.$ For mean distance from the center, results revealed lower mean distance from the center in Nogo-A knockdown rats, *F*(1, 16) = 26.79, *p* = 0.0001, $\eta_{G}^{2}=0.58,$ and no interaction between group and phase factors, *F*(1, 16) = 1.43, *p* = 0.25, $\eta_{G}^{2}=0.01.$ Finally, analysis of defecation showed lower defecation in Nogo-A knockdown rats, *F*(1, 16) = 10.04, *p* = 0.006, $\eta_{G}^{2}=0.32,$ but no interaction between group and phase factors, *F*(1, 16) < 1, *p* = 0.60, $\eta_{G}^{2}=0.00.$

Similar analysis for maximum time avoided with the three rats from each group excluded showed lower maximum time avoided in the Nogo-A knockdown group, *F*(1, 10) = 17.19, *p* = 0.002, $\eta_{G}^{2}=0.27,$ and no interaction between group and phase factors, *F*(1, 10) = 1.07, *p* = 0.33, $\eta_{G}^{2}=0.08.$ Analysis for total distance without the excluded subjects showed neither an effect of group, *F*(1, 10) = 1.20, *p* = 0.30, $\eta_{G}^{2}=0.06,$ nor an interaction between group and phase factors, *F*(1, 10) = 1.78, *p* = 0.21, $\eta_{G}^{2}=0.08.$ Without the excluded subjects, lower mean distance from the center was again observed for the Nogo-A knockdown group, *F*(1, 10) = 24.13, *p* = 0.0006, $\eta_{G}^{2}=0.65,$ while the interaction between group and phase factors was not significant, *F*(1, 10) = 1.44, *p* = 0.26, $\eta_{G}^{2}=0.03$ (Figure 2B). Finally, analysis of defecation after the exclusion of rats again revealed lower defecation in Nogo-A knockdown rats, *F*(1, 10) = 7.24, *p* = 0.02, $\eta_{G}^{2}=0.37,$ and no interaction between group and phase factors, *F*(1, 10) < 1, *p* = 0.81, $\eta_{G}^{2}=0.00$(Figure 2C).

Analysis of proportion of time in the opposite sector revealed no significant difference between groups when done both without, *t*(10.40) = 1.40, *p* = 0.19, *r* = 0.33, and with the exclusion, *t*(6.70) = 1.89, *p* = 0.10, *r* = 0.51. Surprisingly, in both cases, the proportion of time spent in the opposite sector was higher in the Nogo-A knockdown group, which is in contrast to the expectation of higher perseverance (Figure 3B). That the difference did not decrease after the exclusion of subjects shows that it was not caused only by the lower values of the rats displaying freezing behavior.

### Step-through avoidance

**Figure 4:**
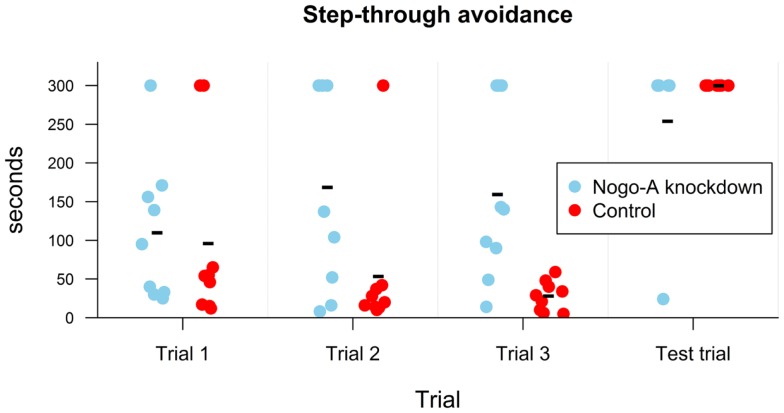
**Results of step-through avoidance in seconds**. Individual points display values for each subject. Group mean is shown with a minus sign. While no difference is present in the first trial, in the subsequent two trials it took longer for Nogo-A knockdown rats to move into the dark compartment. All subjects with the exception of one remained in the light compartment during the entire test trial, i.e., after obtaining a shock in the dark compartment in the third trial (the three Nogo-A knockdown rats, which did not move into the dark compartment during the third trial were not given the test trial since they did not obtain the shock). Maximum possible duration of the trial was 300 s.

A mixed ANOVA for time required to move into the dark compartment showed a significant effect of group, *t*(16) = 2.29, *p* = 0.04, *r* = 0.50, marginally significant linear contrast for trial, *t*(32) = −1.94, *p* = 0.06, *r* = −0.32, and insignificant quadratic contrast for trial, *t*(32) = 0.29, *p* = 0.78, *r* = 0.05. The interaction between linear contrast for trial and group was significant, *t*(32) = 2.37, *p* = 0.02, *r* = 0.38, while the interaction between quadratic contrast for trial and group was not, *t*(32) = −0.99, *p* = 0.33, *r* = −0.17. To explore the interaction between linear contrast for trial and group, we conducted separate repeated measures ANOVAs for both groups. The analyses showed that whereas for the Nogo-A knockdown group the coefficient for linear contrast was positive, although insignificant, *t*(16) = 1.46, *p* = 0.16, *r* = 0.34, it was negative and marginally significant for control group, *t*(16) = −1.88, *p* = 0.08, *r* = −0.42. The interaction can be additionally explored with a separate Welch’s *t*-tests for each trial. While there was no difference between groups for the first trial, *t*(15.13) = 0.28, *p* = 0.78, *r* = 0.07, the Nogo-A knockdown group had higher values in the second, *t*(14.46) = 2.15, *p* = 0.05, *r* = 0.47, and third trials, *t*(8.46) = 3.44, *p* = 0.008, *r* = 0.65 (Figure 4). Therefore, the results showed that time required to move into the dark compartment was the same during the first trial and then decreased for the control group. However, it slightly increased for the Nogo-A knockdown group, which caused a widening difference between both groups during the second and third trial. Both the experimental and control groups exhibited increased latency to enter the dark compartment during the testing session (after the foot-shock), demonstrating successful memory for the unpleasant experience. In fact, only one of the Nogo-A knockdown subjects moved into the dark compartment and none of the control subjects did.

### Neophobia/anhedonia

**Figure 5:**
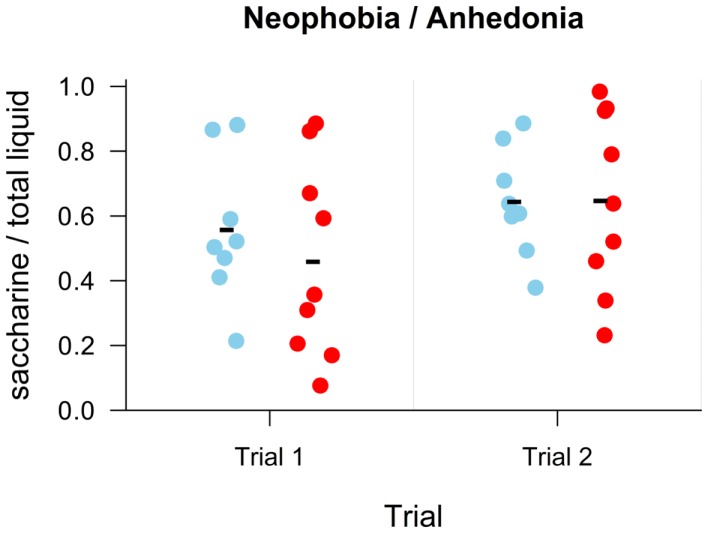
**Results of the neophobia/anhedonia task as a ratio of consumed saccharine to total consumed liquid**. Individual points display values for each subject. Group mean is shown with a minus sign. No difference is seen between groups (blue – Nogo-A knockdown, red – control) in either trial.

All animals behaved as expected during the testing sessions, i.e., tasted and drank the presented liquids. The rats drank slightly more liquid in the second session compared to the first one. For easier comparison, the ratio of consumed saccharine to total amount of liquid consumed was evaluated. We found no effect of group, *F*(1, 15) < 1, *p* = 0.60, $\eta_{G}^{2}=0.01,$ session, *F*(1, 15) = 2.71, *p* = 0.12, $\eta_{G}^{2}=0.08,$ and no effect of interaction between group and session, *F*(1, 15) < 1, *p* = 0.55, $\eta_{G}^{2}=0.01,$ on ratio of consumed saccharine to total amount of liquid consumed (Figure 5).

### Circadian locomotor activity

**Figure 6:**
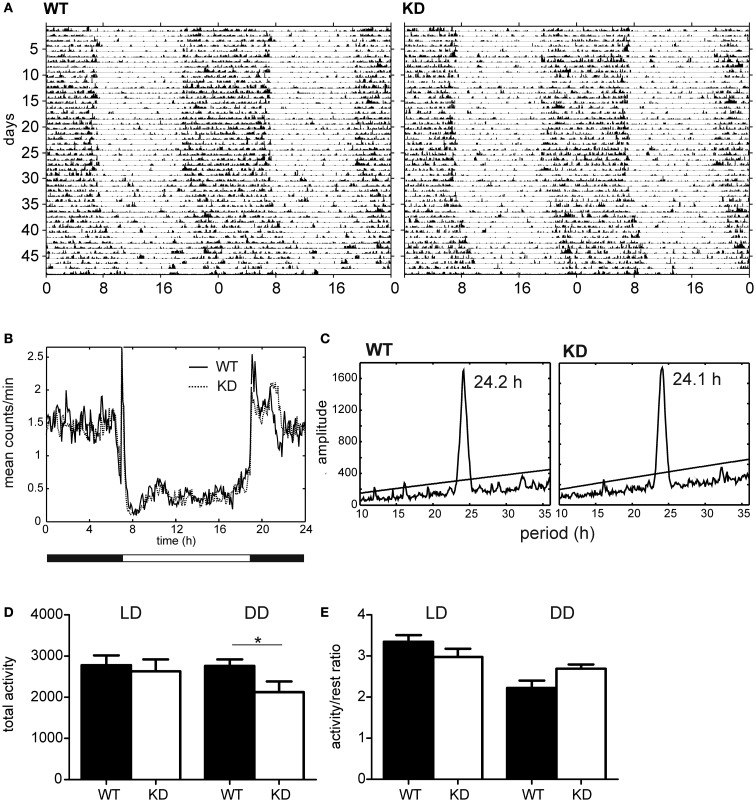
**Activity profiles of Nogo-A knockdown rats (designated KD) and wild-type (WT) controls**. **(A)** Representative double-plotted actograms of locomotor activity of WT (left) and KD (right) rats maintained under a light–dark regime with 12 h of light and 12 h of darkness (LD12:12) for 29 days and subsequently under constant darkness (DD) for 16 days. **(B)** Cumulative locomotor activity profile of eight WT (solid line) and six KD (dashed line) rats kept under LD12:12. **(C)** Cumulative periodogram of eight WT (left) and six KD (right) rats kept under DD. **(D)** Total locomotor activity of eight WT (black) and six KD (white) rats kept under LD12:12 (left) or under DD (right). **(E)** Activity/rest ratio of eight WT (black) and six KD (white) rats kept under LD12:12 (left) or under DD (right).

Spontaneous locomotor activity was monitored continuously in rats maintained under LD12:12 for 1 month and then released into DD for 16 days. The representative activity records (actograms) of one Nogo-A knockdown and one control rat are depicted in Figure 6A. Under LD12:12, the activity exhibited clear daily rhythm with increased levels during the dark phase and decreased levels during the light phase in both rat genotypes. The accumulated activity profiles measured under LD12:12 did not reveal significant differences between the Nogo-A knockdown and WT rats in the phasing or amplitude of the activity levels during the dark and light phases of the light/dark cycle (Figure 6B). Also, total activity (Figure 6D) and activity/rest ratio (Figure 6E) were not significantly different [*t*(11) = 0.39, *p* = 0.70, *r* = 0.12 and *t*(11) = 1.42, *p* = 0.18, *r* = 0.39, respectively] between both rat genotypes maintained under LD cycle. After releasing the rats into constant darkness, the behavioral activity maintained the circadian rhythm, which ran with an endogenous circadian period tau (Figure 6A). Comparison of the endogenous periods tau calculated from the periodograms (Figure 6C) revealed a marginally significant difference (*U* = 7, *p* = 0.05; Mann–Whitney test) between the Nogo-A knockdown (mean ± SD, 24.1 ± 0.1 h, *n* = 5) and WT (24.2 ± 0.1 h, *n* = 8) rats. Under DD, the total activity of the Nogo-A knockdown rats was significantly reduced [*t*(11) = 2.24, *p* = 0.05, *r* = 0.56] (Figure 6D) and there was a trend toward increased activity/rest ratio compared with the controls (Figure 6E) [*t*(11) = 1.93, *p* = 0.08, *r* = 0.50; Student’s *t*-test]. Whereas the controls activity/rest ratio significantly dropped [*t*(14) = 4.64, *p* = 0.0004, *r* = 0.78; Student’s *t*-test] after releasing from LD12:12 into DD, in Nogo-A knockdown, the decline in the ratio was not significant [*t*(8) = 1.23, *p* = 0.25, *r* = 0.40; Student’s *t*-test].

## Discussion

### Nogo-A knockdown is linked to a cognitive deficit in the Carousel maze

During the active place avoidance training in the Carousel maze, both groups of rats exhibited comparable locomotor activity, as measured by total distance (Figure 2A). The Nogo-A knockdown animals performed significantly worse as shown by the lower maximum time avoided. The impairment was slightly (but not significantly) more pronounced during reversal, when the sector position was changed and the animals had to adjust their behavior accordingly (Figure 3A). We might therefore assume that the Nogo-A knockdown rats were impaired in spatial learning and reference frames segregation *per se*, with a possible contribution of behavioral inflexibility. This finding closely parallels that of Petrasek et al. (2014), although the task used here is slightly different (in the present study, the task was purely aversive, without simultaneous foraging).

Reversal learning in the active place avoidance has been recently suggested to tap mnemonic segregation (distinguishing the original learned response, now irrelevant, from the new and relevant), adding another dimension of pattern separation to the already present demand to separate spatial frames (Abdel Baki et al., 2009; Burghardt et al., 2012). It is therefore not surprising that very mild deficits became pronounced during this phase of training. Impairment of reversal learning in a spatial task (water *T*-maze) has been already described in Nogo knockdown mice (Willi et al., 2010) and rats (Tews et al., 2013) and attributed to perseveration. However, in the present study, the Nogo-A knockdown animals exhibited no signs of excessive perseveration, i.e., prolonged avoidance of the no-longer-punished sector. They actually spent *more* time in the former to-be-avoided sector during reversal phase, as shown in Figure 3B.

While our study focused on Nogo-A knockdown, there is a robust body of evidence connecting *facilitated* Nogo-A signaling with impairments of hippocampus-dependent cognitive functions. For example, mice over-expressing NgR show impairment of hippocampus-dependent long-term memory (Karlén et al., 2009). Increased levels of hippocampal Nogo-A showing strong positive correlation with cognitive decline in aged rats (VanGuilder et al., 2011, 2012) and cognitive impairments in a mouse model of Alzheimer’s disease can be ameliorated by genetic deletion of Nogo-A (Masliah et al., 2010). The effects of decreased Nogo-A expression on the behavior of otherwise intact animals are less well explored. In several studies, no behavioral effects were reported after acute blockade of Nogo-A by antibodies in healthy mice (Lenzlinger et al., 2005; Marklund et al., 2007). The permanent absence of Nogo-A in knockout models seems to be more relevant from the neurodevelopmental viewpoint. In some studies, no behavioral effect of knockout has been noted in otherwise intact animals (Marklund et al., 2009; Masliah et al., 2010). In other cases, the deletion of the Nogo-A led to schizophrenia-related endophenotypes, as reported by Willi et al. (2010). The symptoms included disrupted sensorimotor gating and latent inhibition, perseverative behavior in reversal learning, and increased sensitivity to amphetamines. This discrepancy can be explained by differences in the behavioral paradigms employed, as the tasks used by Willi et al. (2010) were specifically chosen to search for schizophrenia-like behavior. Subtle cognitive deficits in Nogo-A knockdown rats have been confirmed by Tews et al. (2013) and our own previous work (Petrasek et al., 2014).

### Nogo-A knockdown decreases anxiety levels

Some of the rats, especially from the control group, showed signs of excessive anxiety or fear during the testing, in some cases resulting in persistent freezing across multiple sessions (“non-solvers”). This behavior has been noted in healthy Sprague-Dawley rats in an active place avoidance task before (the “poor learners” in Carr et al., 2011). As immobile animals are incapable of solving the task, regardless of their cognitive abilities, such animals had to be removed from the analysis. In Nogo-A knockdown rats, passive behavior occurred rarely and never persisted across multiple sessions. Even after the exclusion of “non-solvers,” control animals were apparently more anxious than the Nogo-A knockdown group, as revealed by more pronounced thigmotaxis and increased defecation during avoidance sessions (Figures 2B,C). The difference was apparently present even before introduction of the foot-shock, as the Nogo-A knockdown rats were less thigmotactic and more active than controls even during habituation sessions, when no aversive stimulus was present, except for the novel environment itself. The preferential occurrence of “non-solvers” in the control group was thus presumably linked to the higher anxiety levels, perhaps in a manner similar to learned helplessness. The strong difference in anxiety levels is somewhat surprising, as previous studies both in the rat model (Tews et al., 2013) and in knockout mice (Willi et al., 2009) did not show any difference in anxiety levels.

### Passive avoidance task reveals spared non-spatial memory and normal habituation to a novel taste

In the passive avoidance task, both the experimental and control groups exhibited increased latency to enter the dark compartment during the testing session (after the foot-shock), demonstrating successful memory for the unpleasant stimulus after 24 h. However, their behavior during the first three trials (i.e., without previous experience of the punishment) was different: while control animals entered the preferred dark environment with shorter and shorter latency in subsequent sessions, the Nogo-A knockdown rats exhibited similar or even increased latency with repeated experience, and some of them did not enter it at all (Figure 4). In the context of the Carousel maze results, we can assume that the Nogo-A knockdown rats were less anxious and therefore less motivated to seek shelter in the dark compartment.

The neophobia/anhedonia experiment revealed no difference in taste preferences between the groups (Figure 5).

### Changes in circadian rhythmicity

Nogo-A knockdown rats exhibited daily and circadian rhythms in locomotor activity. Therefore, the ability of the circadian clock to entrain to LD cycle and to drive the circadian rhythms was not affected by the reduction of Nogo-A mRNA and protein levels in retina and brain. Nevertheless, under constant dark conditions, the circadian rhythm in locomotor activity was better expressed in Nogo-A knockdown rats, because the ratio of their activity during the subjective night and subjective day was higher compared with the WT controls. The difference was not due to greater activity in the Nogo-A knockdown rats because their total activity in DD was rather reduced compared with controls. The difference was likely related to the endogenous state of the circadian clock in the SCN, which directly drives the activity rhythm in constant darkness, because the difference between both phenotypes was not apparent in LD conditions, when the activity was regulated by the circadian clock as well as directly suppressed by light via masking effect (Figure 6). Therefore, the data suggest that the partial developmental deficit in Nogo-A may modulate the clock function. It is, however, not known whether the modulation arises from changes of the clock property *per se* or from the fact that the input neural pathways from other brain areas may be modulated by the Nogo-A deficiency. Also, an impact of the genotype on the sensitivity of the locomotor activity to LD cycle cannot be completely ruled out because in mice with complete deletion of Nogo-A activity during the dark phase of the LD cycle was higher than in WT controls. The activity during the light phase was not different, which means that the activity/rest ratio in the Nogo-A knockout mice was increased even under LD cycle (Willi et al., 2009).

The aforementioned data suggest that the circadian clock in Nogo-A knockdown might function as a “stronger” circadian pacemaker than that of WT rats. As the robustness of the circadian clock in the SCN is highly dependent on the synaptic communication among the individual oscillators (Liu et al., 2007), the reduction of the neurite outgrowth inhibitor during development in Nogo-A knockdown rats might theoretically be beneficial for the clock function. However, this highly speculative conclusion would need to be verified in future experiments comparing the synaptic strength and amplitudes of clock gene and protein expression level profiles directly in the SCN between the Nogo-A knockdown and WT control rats.

### Caveats

Because of the necessary exclusions, the number of animals used in this work was not large, raising concerns about power of the statistical tests used. However, in some measures, the difference between the two groups was large enough to be detected unambiguously.

Another caveat is that the Nogo-A knockdown group consisted of two litters. While we do not believe that this seriously affected the results, it should be taken into account in interpreting the results.

### Nogo-A knockdown as a model of schizophrenia

In mouse and rat models, Nogo-A-deficient animals exhibit symptoms such as disrupted sensorimotor gating and latent inhibition, deficits of memory, cognitive flexibility, and social behavior (Willi et al., 2010; Tews et al., 2013; Petrasek et al., 2014, the present work), which are characteristic for schizophrenia models, and are considered analogous to cognitive and negative symptoms in humans (Bubenikova-Valesova et al., 2008b; Jones et al., 2011). As disruption of Nogo-A signaling may be relevant at least in some cases of human schizophrenia pathogenesis (Willi and Schwab, 2013), this model can exhibit construct validity as well.

Hyperlocomotion and stereotypic behavior are considered animal analogs to positive (psychotic) symptoms (Bubenikova-Valesova et al., 2008b). In the Nogo-A-deficient models, changes in locomotor behavior are rather subtle and reported only in some experimental settings, while stereotypies (e.g., stereotypic grooming) have not been observed. This might indicate that the Nogo-A knockout/knockdown induces alterations similar to negative and cognitive, but not positive schizophrenia symptoms. Differential expression of symptoms, often with some classes less pronounced or entirely missing, is rather typical for animal models of schizophrenia, as well as the disease itself in human patients. Preferential expression of negative and cognitive symptoms in an animal model might be even viewed as an advantage, as these classes are rather under-represented in the traditional models of the disease (Jones et al., 2011).

Our results from the present work and Petrasek et al. (2014) demonstrate mostly cognitive deficits specific for Carousel maze tasks requiring spatial frames segregation and cognitive flexibility, which is consistent with schizophrenia-like symptomatology, as can be demonstrated in comparison with similar studies using different models. Ample experimental data from the Carousel maze tests have been collected using pharmacological dizocilpine (MK-801) model of schizophrenia (Stuchlik et al., 2004; Vales et al., 2006; Bubenikova-Valesova et al., 2008a). Dizocilpine can disrupt Moris Water Maze performance even before Carousel maze performance (Stuchlik et al., 2004), unlike Nogo-A knockdown model, where Water Maze learning is intact (Petrasek et al., 2014). On the other hand, dizocilpine administration leads to deficits in spatial reversal learning (Lobellova et al., 2013), which is similar to Nogo-A deficiency.

Neurodevelopmental models of schizophrenia should be perhaps more comparable to the Nogo-A knockdown and knockout models than acute pharmacological treatments, but their influence on Carousel maze performance is less well studied. An exception is the neonatal ventral hippocampal lesion (NVHL) model (Lecourtier et al., 2012; Lee et al., 2012; Swerdlow et al., 2012). Lee et al. (2012) have found that the NVHL rats are impaired in the Carousel maze learning, and even more in reversal learning, which parallels our findings in Nogo-A knockdown rats.

We must note that there is no “ideal” animal model of schizophrenia. Etiology of schizophrenia is largely unknown (and probably multi-factorial), and all we can reasonably assess is the similarity of symptoms (face validity). Furthermore, there is no unambiguous biochemical, anatomical, pharmacological, or behavioral marker of schizophrenia that could be used to reliably validate proposed animal models (Lipska and Weinberger, 2000). From this perspective, Nogo-A-deficient transgenic animals constitute a novel candidate model of schizophrenia with proposed construct validity and good face validity at least for negative and cognitive symptoms.

### Summary

Results of the present study clearly demonstrate behavioral differences between the rats with decreased Nogo-A expression and WT Sprague-Dawley controls. We can conclude that the Nogo-A knockdown rats exhibited marked cognitive deficit in the Carousel maze. In spite of their reduced ability to avoid punishment, they seemed less anxious than their WT counterparts. Non-spatial long-term memory, assessed in the passive avoidance task, and neophobic reaction to a novel taste and preference of a sweet taste did not differ between groups. The period of circadian rhythms was not affected, but in constant darkness, the rhythm in locomotor activity was more pronounced in the Nogo-A knockdown animals.

When taken together with the biochemical assessment of key protein expression and laterality in the brain of the same transgenic rat line (Krištofiková et al., 2013), our results are mostly consistent with the proposed link between decreased Nogo-A levels and schizophrenia-like behavior in the rat, even though some of the results suggest that changes caused by Nogo-A knockdown are more complex than previously thought.

## Conflict of Interest Statement

The authors declare that the research was conducted in the absence of any commercial or financial relationships that could be construed as a potential conflict of interest.
