# Corrigendum: Nogo-A-deficient transgenic rats show deficits in higher cognitive functions, decreased anxiety, and altered circadian activity patterns

**DOI:** 10.3389/fnbeh.2014.00442

**Published:** 2014-12-23

**Authors:** Tomas Petrasek, Iva Prokopova, Martin Sladek, Kamila Weissova, Iveta Vojtechova, Štěpán Bahník, Anna Zemanova, Kai Schonig, Stefan Berger, Bjorn Tews, Dusan Bartsch, Martin E. Schwab, Alena Sumova, Ales Stuchlik

**Affiliations:** ^1^Department of Neurophysiology of Memory, Institute of Physiology, Academy of Sciences of the Czech RepublicPrague, Czech Republic; ^2^First Faculty of Medicine, Charles University in PraguePrague, Czech Republic; ^3^Department of Neurohumoral Regulations, Institute of Physiology, Academy of Sciences of the Czech RepublicPrague, Czech Republic; ^4^Department of Psychology II, Social Psychology, University of WürzburgWürzburg, Germany; ^5^Department of Molecular Biology, Central Institute of Mental HealthMannheim, Germany; ^6^Brain Research Institute, University of Zürich, and Department of Biology, ETH ZürichZürich, Switzerland; ^7^Division of Molecular Mechanisms of Tumor Invasion, German Cancer Research CenterHeidelberg, Germany

**Keywords:** corrigendum, Nogo-A, behavior, animal, circadian rhythm, memory, navigation, anxiety

In the paper by Petrasek et al., a mistake was made in the Acknowledgments Section. The corrected Acknowledgments follow:

## Conflict of interest statement

The authors declare that the research was conducted in the absence of any commercial or financial relationships that could be construed as a potential conflict of interest.

